# Alterations in Nitric Oxide Activity and Sensitivity in Early Streptozotocin-Induced Diabetes Depend on Arteriolar Size

**DOI:** 10.1155/EDR.2000.221

**Published:** 2000

**Authors:** Bastiaan vanDam, Cihan Demirci, Hans J. Reitsma, Anton A. van Lambalgen, Gerard C. van den Bos, Geert Jan Tangelder, Coen D. A. Stehouwer

**Affiliations:** 1 Department of Internal Medicine Institute for Cardiovascular Research Vrije Universiteit The Netherlands; 2 Department of Biology Istanbul Üniversitesi Istanbul Turkey; 3 Laboratory for Physiology Institute for Cardiovascular Research Vrije Universiteit Amsterdam The Netherlands

## Abstract

Changes in NO activity may play an important role in the early increase in microvascular flow that has been implicated in the pathogenesis of diabetic microangiopathy. We assessed, in the *in situ* spinotrapezius muscle preparation of 6 weeks' streptozotocin-diabetic rats (*n* = 6) and of agematched controls (*n* = 8), basal inside diameters of A2–A4 arterioles and the reactivity to topically applied acetylcholine and nitroprusside, before and after N^G^-nitro-L-arginine. In diabetic rats, cholinergic vasodilatation in A2–A4 arterioles was intact. Basal diameter in A3 and A4 arterioles was significantly higher in streptozotocin-diabetic rats. The increased basal diameter in A3 arterioles was partially due to an increased contribution of NO to basal diameter. The response to nitroprusside was impaired in streptozotocin-diabetic rats in A2, but not in A3 and A4 arterioles. Thus, this study shows that NO activity and sensitivity are altered after 6 weeks of streptozotocin-induced diabetes. These streptozotocin-induced changes are anatomically specific and, for arterioles, depend on their position within the vascular tree.


*Int. Jnl. Experimental Diab. Res.*, Vol. 1, pp. 221–232
Reprints available directly from the publisher
Photocopying permitted by license only

© 2000 OPA (Overseas Publishers Association) N.V.
Published by license under
the Harwood Academic Publishers imprint,
part of The Gordon and Breach Publishing Group.
Printed in Malaysia.

### Alterations in Nitric Oxide Activity and Sensitivity in Early Streptozotocin-induced Diabetes Depend on Arteriolar Size

BASTIAAN VAN DAM[a], CIHAN DEMIRCI[b], HANS J. REITSMA[c], ANTON A. VAN LAMBALGEN[c], GERARD C. VAN DEN BOS[c], GEERT JAN TANGELDER[c] and COEN D. A. STEHOUWER[a,*]

[a]*Department of Internal Medicine, Institute for Cardiovascular Research, Vrije Universiteit;* [b]*Department of Biology, Istanbul Üniversitesi, Istanbul, Turkey;* [c]*Laboratory for Physiology, Institute for Cardiovascular Research, Vrije Universiteit, Amsterdam, The Netherlands*

*(Received 6 December 1999; Revised 5 April 2000; In final form 11 May 2000)*

**Changes in NO activity may play an important role in the early increase in microvascular flow that has been implicated in the pathogenesis of diabetic microangiopathy. We assessed, in the *in situ* spinotrapezius muscle preparation of 6 weeks' streptozotocin-diabetic rats ($n = 6$) and of age-matched controls ($n = 8$), basal inside diameters of A2–A4 arterioles and the reactivity to topically applied acetylcholine and nitroprusside, before and after N$^G$-nitro-L-arginine. In diabetic rats, cholinergic vasodilatation in A2–A4 arterioles was intact. Basal diameter in A3 and A4 arterioles was significantly higher in streptozotocin-diabetic rats. The increased basal diameter in A3 arterioles was partially due to an increased contribution of NO to basal diameter. The response to nitroprusside was impaired in streptozotocin-diabetic rats in A2, but not in A3 and A4 arterioles. Thus, this study shows that NO activity and sensitivity are altered after 6 weeks of streptozotocin-induced diabetes. These streptozotocin-induced changes are anatomically specific and, for arterioles, depend on their position within the vascular tree.**

*Keywords*: Hyperglycemia; Microcirculation; Streptozotocin; Acetylcholine; Nitroprusside; Nitric oxide; Diabetes mellitus

### INTRODUCTION

Microangiopathy has a major impact on morbidity and mortality of patients with diabetes mellitus.[1] An important hypothesis regarding the pathophysiology of diabetic microangiopathy is the hemodynamic hypothesis,[2] which states that an early, reversible increase in microvascular flow eventually leads to an endothelial injury response and microvascular sclerosis.[2–4]

Using a mathematical model and experimental data on nitric oxide (NO) production and degradation rates, Vaughn *et al.*, showed that the range of NO action may exhibit significant spatial heterogeneity *in vivo* and that the microcirculation is the optimal site for NO to exert its regulatory function.[5,6] An increased flow early in diabetes, possibly as a result of enhanced NO production, has been described in several microvascular beds.[7,8] However, those studies

*Corresponding author. Department of Internal Medicine, De Boelelaan 1117, 1081 HV Amsterdam, The Netherlands. Tel.: +31 20 4440629/4440531, Fax: +31 20 4440502, e-mail: cda.stehouwer@azvu.nl addressed only overall changes in flow. In order to gain insight in the exact role of NO in microangiopathy, it is necessary to assess its activity and effectivity at different sites of the microcirculation, as several orders of arterioles can react differently to chemical and mechanical vasoactive stimuli.[9–11] Recently, it was shown that the small arterioles that control capillary perfusion are capable of responding differently in a spatially organised way and that metabolic changes are able to change this pattern of diameter change.[12]

The earliest changes in the microvasculature caused by the metabolic disturbance in type 1 diabetes can most likely be observed in striated muscle, which is metabolically the most active tissue. Indeed, changes in reactions in different orders of arterioles of striated muscle to several vasoactive substances have been observed in rats 6 weeks after the induction of diabetes by streptozotocin (STZ).[13]

To gain more insight regarding the role of NO in the pathophysiology of diabetes-induced microangiopathy, we investigated its contribution to vascular tone in arcade (A2) arterioles and the smaller transverse (A3) and precapillary (A4) arterioles in the *in situ* spinotrapezius muscle preparation[14,15] of control rats and rats with STZ-induced diabetes. We chose the spinotrapezius muscle because it is easily accessible without much trauma, because different orders of arterioles can be studied with intravital microscopy, and because drugs can be applied topically, thus preventing systemic changes.

The aim of the present study, therefore, was to assess, *in vivo*, the contribution of NO to basal tone and the reactivity to nitroprusside in different orders of arterioles after 6 weeks of STZ-induced diabetes, a widely studied model of type 1 diabetes.[16]

### MATERIAL AND METHODS

### Experimental Animals

All experiments were performed according to the NIH guidelines for the use of experimental animals (Helsinki declaration), after permission of the institutional animal care and use committee. Diabetes was induced in 6 conscious male Wistar rats (~250 g; HSD, Zeist, The Netherlands) by a single tail vein injection of 65 mg/kg STZ dissolved in sodium citrate buffer (pH = 4.5). Glucose levels were assessed weekly in each animal with a haemo-glukotest (Accutrend Alpha, Boehringer Mannheim) in blood samples taken from the tail tip. The animals were housed in individual cages and were allowed free access to water and pre-weighed commercial rat pellet chow. Experiments were performed 6 weeks after administration of STZ. Untreated male Wistar rats ($n = 8$) were used as controls.

### General Set Up

Anesthesia was induced in overnight fasted rats by an injection of pentobarbital sodium (Nembutal$^R$; 60 mg/kg, intraperitoneally) and ketamine (70 mg/kg, intramuscularly). For further administration of Nembutal$^R$, an intraperitoneal cannula was inserted. As an indication of the need for additional small doses of the anesthetic (25% of the original dose), we frequently monitored blood pressure, interdigital and eye reflexes, as well as respiration rate with the intent of keeping the depth of the anesthesia stable. In our hands, this anesthetic protocol caused stable hemodynamics and blood gas values throughout the entire experimental period (approximately 5 hrs), indicating a constancy of the anesthetic conditions. Moreover, in previous studies[17,18] we have also found a stable $O_2$-consumption of the animal and low blood lactate levels throughout the whole experiment. During the experiment, body temperature was kept at 37–38°C by a heating pad placed under the rat. The trachea was cannulated to maintain a patent airway.

To be informed about the systemic hemodynamic variables which could influence the microcirculation we measured cardiac output in addition to blood pressure. Mean arterial pressure (MAP) was determined with a catheter

(PE50) placed in the right carotid artery. The heart rate (HR) was obtained from the blood pressure recording. Cardiac output (CO) was measured by thermodilution: saline (0.2 ml) at room temperature was injected from a catheter that was inserted in the right jugular vein, with the tip near the right atrium. A thermistor, which was obtained from a Swan–Ganz catheter (Gould, Cleveland, OH), was inserted into the abdominal aorta *via* a femoral artery. The thermistor was connected to an Edwards 9520A (Santa Ana, CA) cardiac output computer.

Arterial blood samples were taken from a carotid artery at the beginning and the end of an experiment to measure glucose and insulin (by radioimmunoassay; Immunoradiometric Assay, Medgenic Diagnostics, Fleurus, Belgium).

### Spinotrapezius Preparation

The left spinotrapezius muscle was approached through a skin incision along the spine, after which subcutaneous fat and fascia were carefully removed. The lateral border of the muscle was freed and lifted with 4 to 5 atraumatic sutures to separate it from the underlying muscle layers. It was then draped, with the ventral surface upward, over a perspex pedestal placed on the microscope stage (Fig. 1) as described by Gray [14] and Marshall. [19] A specific area of interest was selected at the caudo-medial side in which different orders of arterioles were present.

Classification of the vessels [20, 21] is also illustrated in Figure 1. Vessels derived directly from feeding or A1 arterioles and forming main arcades are considered A2 arterioles. Vessels derived from these larger arcade vessels, forming smaller arcades, are considered A3 arterioles. Finally, as A4 arterioles we considered vessels that supply blood from the smallest arcades to terminal arterioles.

The muscle was superfused with tyrode solution (NaCl, 128.3, KCl 4.7, $CaCl_2 \cdot H_2O$ 1.36, $MgCl_2 \cdot 6H_2O$ 1.05, $NaHCO_3$ 20.2, $NaH_2PO_4 \cdot 2H_2O$ 0.42 mol/L). During the experiment the superfusate was equilibrated with 95% $N_2$ and 5% $CO_2$, to prevent direct oxygenation of the muscle cells by high levels of oxygen in the surrounding fluid. Only during the dissection was this solution equilibrated with 95% $O_2$ and 5% $CO_2$. The temperature of the solution was maintained at ~36°C at the muscle surface; the pH was 7.4.

We used a Zeiss microscope (Zeiss, Germany) with a 25xSW objective (numerical aperture 0.63; Leitz, Germany). The optical magnification at the front plane of the video camera was 58.8x. The experiments were recorded on video tape (Panasonic AG-6200, Japan) using a black and white Philips LDH 0702/20 camera (Philips, The Netherlands). Inside diameters of A2–A4 arterioles were measured on the monitor from stopped images with an interactive computer program (made in our own workshop). [22]

### Protocol

After catheterisation of the rat and preparation of the spinotrapezius muscle had been finished, we moved the animal to the microscope. After a suitable arcade had been found, we waited at least 30 min for stabilisation of the preparation and then started the video microscopic measurements according to the following protocol.

First, the basal inside diameter was assessed. After that, several drugs were added to the superfusate, reaching their final concentrations at the surface of muscle. They were applied until their effect was maximal (usually for 5 min). After the diameter had returned to the control value (after 5–15 min), the next concentration of a drug was started. The arteriolar inside diameters were recorded before, during, and after the entire period of superfusion of the drugs.

The integrity of the endothelial vasodilator response was assessed by testing the effect of ACh. The experimental set up did not allow us to perform a complete dose-response relationship of ACh. Therefore, we focused on the capacity of ACh to dilate the vessels maximally. We used 3 different doses ($10^{-5}$, $10^{-4}$ and $10^{-3}$ mol/L), giving submaximal or maximal

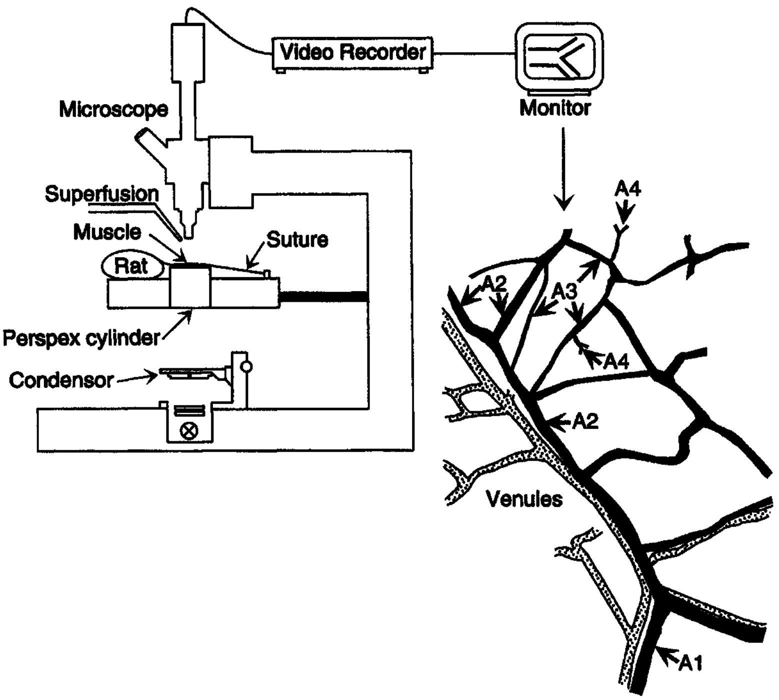

FIGURE 1 *Experimental set-up of the spinotrapezius preparation.* The spinotrapezius muscle is draped over a perspex cylinder positioned in the microscope stage. The dorso-medial side of the muscle, with intact feeding arterioles, remains connected to the rat, which lies on its side. The preparation is superfused with Tyrode solution. The microscope is equipped with a video system to continuously record microvessels on tape. A1: feeding arteriole; A2: main arcade arterioles ( $> 40\,\mu m$); A3: smaller arcade arterioles (2040 µm); A4: arterioles supplying the terminal arterioles (1020 µm).

responses, to accurately assess the level of the maximal response and the dose at which the maximal response was reached.

After we had tested the effect of ACh, we administered the NO-generating endothelium-independent vasodilator nitroprusside (NP: $10^{-4}$ mol/L, *i.e.*, a dose high enough to cause maximal dilatation in control animals, as established in pilot experiments). Finally, adenosine (Ad), a mainly endothelium-independent vasodilator was added.[23–25] The dose of Ad to obtain maximal dilatation was previously assessed at $10^{-4}$ mol/L; the diameter after application of this dose was considered as the passive diameter and this diameter was set at 100%. This approach enabled us to relate basal diameters and the effects of the different drugs to a diameter that was independent from the initial contractile state.[26]

After this series, we added L-NNA ($10^{-4}$ mol/L) to the superfusate to block NO production. Twenty minutes after the start of L-NNA, inside diameter was assessed. The contribution of NO activity to basal diameter was defined as the percentage reduction in basal diameter that could be achieved by L-NNA ($10^{-4}$ mol/L). We repeated the protocol with the dilatory drugs while L-NNA superfusion continued.

### Analysis and Statistics

For statistical evaluation, we used the average values of all A2, A3, or A4 arterioles (ranging from 2 to 10) per rat. In 1 STZ rat muscle preparation no A3 arterioles were evaluated; the number of observations for statistical analysis was thus 6 for the STZ-treated rats in A2 and A4 arterioles, 5 for the STZ-treated rats in the A3

arterioles and 8 for the control group. We used two-tailed non-parametric tests with level of significance set at 5%.

### RESULTS

### General Characteristics

Blood glucose level measured 1 week after the STZ injection was $20 \pm 5$ mmol/L and remained so during the entire period of 6 weeks. Food consumption in DM rats, compared with control rats, almost doubled ($\sim 150$ *vs.* $\sim 300$ g/wk). Nevertheless, the body weight of DM rats remained less than that of the control counterparts ($242 \pm 32$ g *vs.* $350 \pm 36$ g for 6 weeks' diabetic rats and control rats, respectively; $P < 0.05$). There was no relationship between body weight and either passive diameter, baseline diameter or diameter during L-NNA in the different arterioles (data not shown). Systemic hemodynamic and biochemical variables at the start and at the end of the experiments are presented in Table I. At the day of the experiment, after an overnight fast, the glucose levels of both the DM and control rats had been decreased by $\sim 40\%$ as compared to the non-fasted state. Baseline blood pressure was not significantly different between the groups, but heart rate and cardiac output were significantly higher in control rats. Hct was not significantly different between control and diabetic rats. During the actual microcirculation experiments (lasting $\sim 3$ hr), there was a decrease in CO of about 15% in both control and diabetic rats. MAP and HR did not change significantly during the experiments (Tab. I).

TABLE I Systemic variables 24 hr before the experiments ($t = -24$ hr), at the start of the experiments ($t = 0$) and at the end of the experiments ($t = 3$ hr) in control and diabetic rats (DM; six weeks after injection of STZ). Hct: hematocrit; MAP: mean arterial pressure; HR: heart rate; CO: cardiac output. Data are expressed as mean ± SD

| | | Control ($n = 8$) | DM ($n = 6$) |
|---|---|---|---|
| Gluc (mmol/L) | $t = -24$ hr | 5.4 ± 1.1 | 19.9 ± 1.9* |
| | $t = 0$ hr | 4.4 ± 0.5 | 11.2 ± 2.0* |
| | $t = 3$ hr | 4.5 ± 0.6 | 10.4 ± 3.5* |
| Insulin (mU/L) | $t = 0$ hr | 20.6 ± 2.0 | 8.0 ± 2.0* |
| Hct | $t = 0$ hr | 49.7 ± 5.8 | 52.8 ± 2.8 |
| MAP (mmHg) | $t = 0$ hr | 116 ± 17 | 112 ± 15 |
| | $t = 3$ hr | 123 ± 20 | 124 ± 9 |
| HR (bts/min) | $t = 0$ hr | 395 ± 44 | 340 ± 38* |
| | $t = 3$ hr | 400 ± 24 | 347 ± 27* |
| CO (mL/min) | $t = 0$ hr | 98 ± 15 | 79 ± 14* |
| | $t = 3$ hr | 85 ± 12 | 64 ± 10* |

*$P < 0.05$ for DM *vs.* control.

### Inside Arteriolar Diameter in Different Orders of Arterioles

As compared to control rats, DM rats had significantly increased inside diameters of A3 and A4, but not of A2 arterioles in the basal state (Fig. 2, Tab. II). Basal diameters relative to passive diameter did not differ significantly between DM and control rats (Fig. 2).

### Contribution of Endogenous NO to Basal Inside Diameter

Overall, superfusion of L-NNA ($10^{-4}$ mol/L) caused a decrease in basal diameter in both control and diabetic rats (Tab. II), confirming the role of NO in maintaining basal diameters in this model. After blockade of the endogenous NO production, inside arteriolar diameters were not significantly different in DM rats as compared with control rats (Tab. II). However, the contribution of NO activity to basal inside diameter was significantly higher in diabetic rats than in control rats in A3 arterioles (Fig. 3).

### Response to Nitroprusside

After addition of nitroprusside in the basal state, a significantly higher inside diameter was found in all types of arterioles in both control and diabetic rats (Tab. II). However, this increase was smaller in A2 arterioles of diabetic rats as

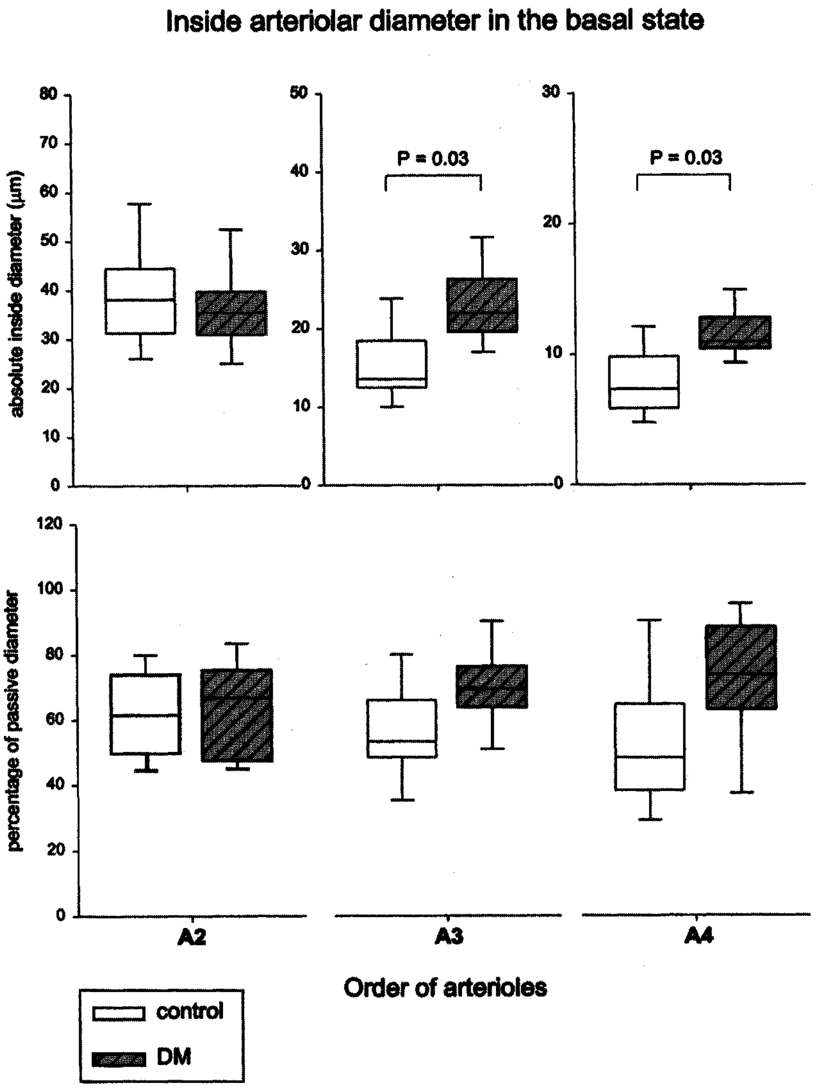

FIGURE 2 *Inside arteriolar diameter in the basal state.* Inside diameters, both absolute (µm) and expressed as percentage of the passive diameter (*i.e.*, after $10^{-4}$ mol/L adenosine; set at 100%), of A2A4 arterioles in the spinotrapezius muscle at baseline in control (open plots) and diabetic rats (DM; filled plots). The data are expressed as box-and-whisker plots: the central line is the median, the lower and upper quartiles are indicated by the box and the 2.5 and 97.5 centiles are indicated by whiskers. *P*-values for the comparison between DM and control rats for absolute basal inside diameters: A2, $P = 0.85$; A3, $P = 0.03$; A4, $P = 0.03$. *P*-values for the comparison between DM and control rats for relative inside diameters: A2, $P = 0.85$; A3, $P = 0.22$; A4, $P = 0.14$.

compared to controls. By contrast, in A3 arterioles inside diameter remained higher under nitroprusside in diabetic animals as compared to controls, while in A4 arterioles similar inside diameters were found (Tab. II). In order to correct for differences in basal NO production, responses to nitroprusside were also assessed after addition of L-NNA (Fig. 4). In contrast to control rats, in DM rats the response to $10^{-4}$ mol/L nitroprusside superfusion was significantly lower in A2, but not in the A3 and A4 arterioles, when endogenous NO production was blocked by L-NNA. We have tested in 3 additional experiments whether higher doses

TABLE II Inside diameters (in μm) of A2, A3 and A4 arterioles in the spinotrapezius of control and diabetic (DM) rats at baseline, after blocking the NO production with L-NNA, and after dilatation with $10^{-4}$M nitroprusside (NP), $10^{-3}$M acetylcholine (ACh) or $10^{-4}$M adenosine (Ad). Data are expressed as mean ± SD

| Order of arteriole | Condition | Control ($n = 8$) | DM ($n = 6$) |
|---|---|---|---|
| A2 | basal | 39.1 ± 11.7 | 36.5 ± 10.1 |
| | L-NNA $10^{-4}$M | 31.6 ± 9.3 | 24.2 ± 4.5 |
| | NP $10^{-4}$M | 61.8 ± 11.7 | 48.9 ± 4.7* |
| | ACh $10^{-3}$M | 62.4 ± 14.1 | 54.5 ± 8.9 |
| | Ad $10^{-4}$M | 63.7 ± 11.8 | 56.4 ± 6.4 |
| A3 | basal | 15.5 ± 5.2 | 23.1 ± 5.5* |
| | L-NNA $10^{-4}$M | 12.3 ± 3.1 | 13.6 ± 2.4 |
| | NP $10^{-4}$M | 26.2 ± 4.1 | 31.6 ± 3.7* |
| | Ach $10^{-3}$M | 25.4 ± 5.8 | 32.3 ± 3.0* |
| | Ad $10^{-4}$M | 27.7 ± 3.5 | 32.8 ± 2.2* |
| A4 | basal | 7.9 ± 2.8 | 11.6 ± 2.1* |
| | L-NNA $10^{-4}$M | 6.2 ± 2.7 | 7.3 ± 3.7 |
| | NP $10^{-4}$M | 15.0 ± 3.1 | 16.7 ± 4.4 |
| | Ach $10^{-3}$M | 15.2 ± 3.4 | 15.9 ± 2.8 |
| | Ad $10^{-4}$M | 15.4 ± 3.4 | 18.0 ± 7.4 |

*$P < 0.05$ for DM *vs.* control.

of NP could further increase the arteriolar diameters in DM rats, but this was not the case (data not shown). Superfusion of higher doses of NP, however, had a complicating disadvantage, because at these doses the NP caused systemic effects (*i.e.*, a decrease in blood pressure).

### Responses to Acetylcholine and Adenosine

Acetylcholine, as compared with adenosine, was able to cause maximal dilatation in both control and diabetic rats in all orders of arterioles. No differences were found between acetylcholine doses of $10^{-4}$ and $10^{-3}$mol/L. In A3 arterioles, inside diameters were significantly higher in diabetic rats after both after acetylcholine and adenosine (Tab. II). However, when endogenous NO production was blocked by L-NNA, inside diameters after adenosine or acetylcholine were

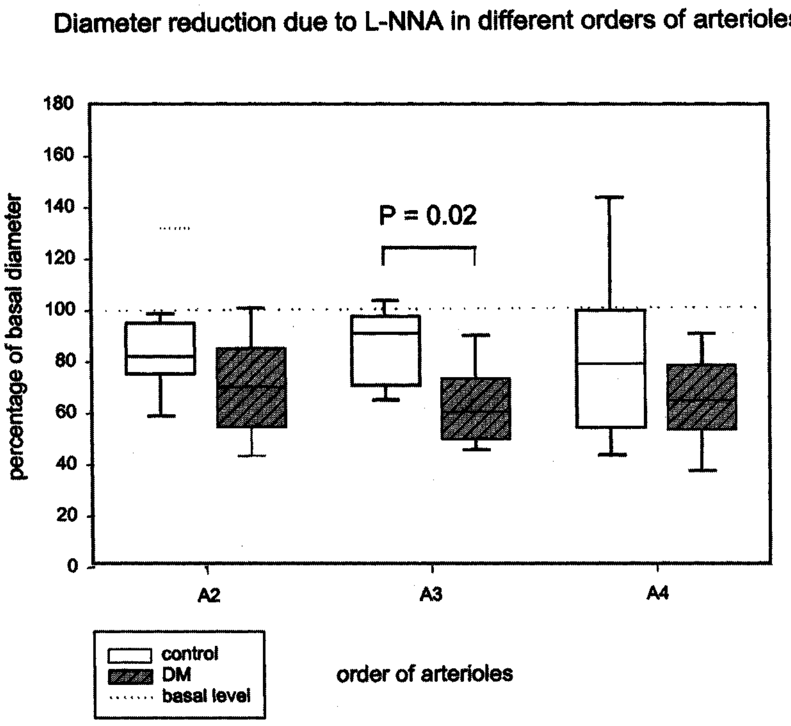

FIGURE 3 *Contribution of endogenous NO to basal inside diameter in different orders of arterioles.* Percentage reduction of the basal diameter of A2A4 arterioles in the spinotrapezius muscle caused by superfusion of L-NNA ($10^{-4}$mol/L) in control (open plots) and in diabetic rats (DM; filled plots); box-and-whisker plots. *P*-values for the comparison between DM and control rats for percentage of basal diameter blockable with L-NNA ($10^{-4}$mol/L): A2, $P = 0.35$; A3, $P = 0.02$; A4, $P = 0.49$.

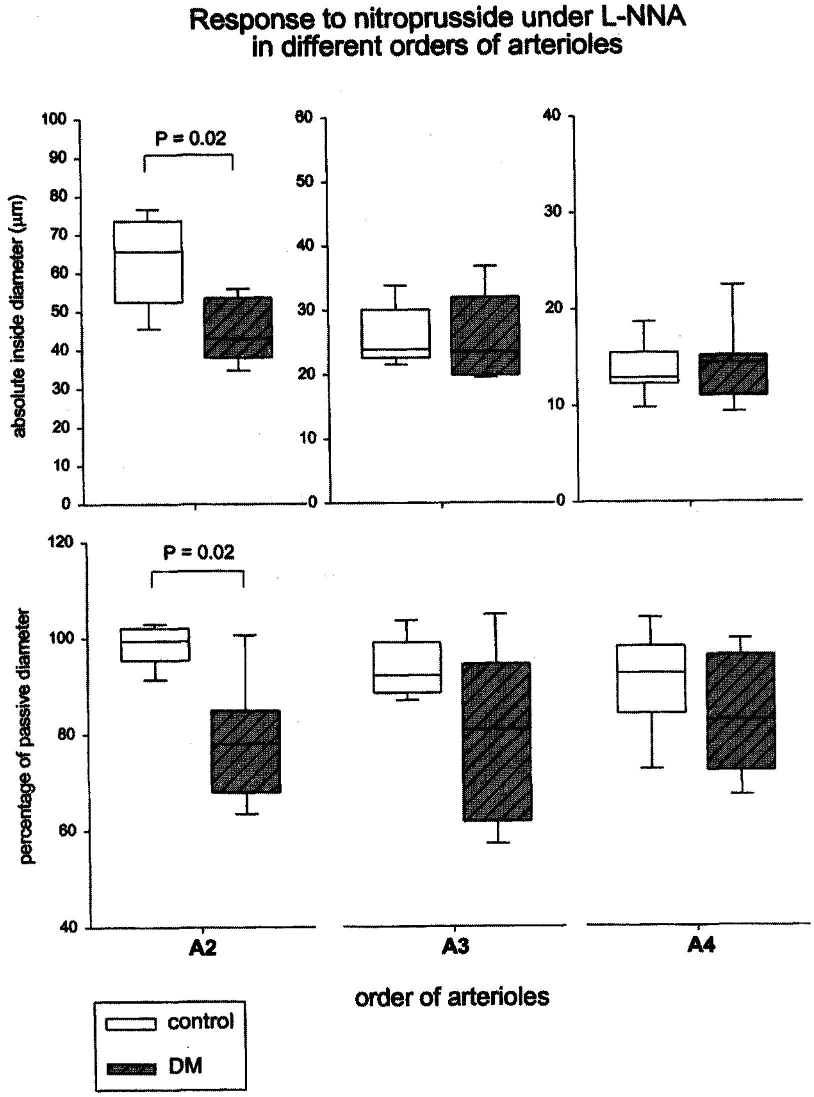

FIGURE 4 *Response to exogenous NO in different orders of arterioles.* Inside diameters, both expressed as absolute diameters (µm) and as percentage of the passive diameter (*i.e.*, after $10^{-4}$mol/L adenosine; set at 100%), of A2A4 arterioles in the spinotrapezius muscle after superfusion of $10^{-4}$mol/l NP during blockade of NO production with L-NNA ($10^{-4}$mol/L) in control (open plots) and diabetic rats (DM; filled plots); box-and-whisker plots. *P*-values for the comparison between DM and control rats for absolute inside diameters: A2, $P = 0.02$; A3, $P = 0.83$; A4, $P = 0.41$. *P*-values for the comparison between DM and control rats for relative inside diameters: A2, $P = 0.02$; A3, $P = 0.17$; A4, $P = 0.66$.

no longer significantly different between diabetic and control rats in these arterioles (data not shown), suggesting that an increased basal production of NO could still cause a further increase in diameter in diabetic rats after either adenosine or acetylcholine.

### DISCUSSION

Induction of diabetes mellitus by streptozotocin treatment in rats is an accepted model of human type 1 diabetes.[16] This model allowed us to investigate differential microcirculatory changes in DM, which cannot be studied in man. We intended and achieved incomplete destruction of pancreatic beta-cells and therefore the diabetic rats had some residual insulin production and thus a comparatively moderate hyperglycemia (~20 mmol/l).[15,27]

Using intravital microscopy, we found an increase in basal diameter in A3 and A4, but not in A2 arterioles after 6 weeks of STZ-induced diabetes mellitus (Fig. 2). There was a greater change in diameter after L-NNA in the diabetic rats as compared to the control rats, especially in A3 arterioles (Fig. 3). This suggests that the increase in basal diameter observed in diabetic rats is at least partially due to an increased contribution of endogenous NO to basal diameter in the A3 arterioles. Although NO production was increased significantly in A3 arterioles only, it cannot be excluded that also in A2 and A4 arterioles there was some increase in NO production in diabetic rats. These findings support the hypothesis of Williamson *et al.*, that an increased NO production occurs in the early phase of DM.[28] An increased NO production was also observed in the kidney of STZ rats.[29] However, several studies also report weaker relaxation responses to endothelium-dependentagents after induction of diabetes with STZ.[30–33] These differences might be explained by variations in experimental set-up, the type of endothelium-dependent relaxation response that was investigated, the type of vessel, the duration of diabetes and differences in the elevation in glucose levels that were obtained in STZ rats.

Although NO has been indicated as an anti-atherosclerotic molecule,[34,35] several studies provide evidence that the increase in NO activity we observed in the early phase of STZ-induced diabetes may be a mediator of vascular damage. An increased NO synthase activity can induce chemical injury to the vessel wall *via* the production of peroxynitrite, a toxic radical.[36–39] An enhanced NO activity in the early phase of diabetes which preceded impaired function in the later phase was described by Pieper *et al.*[38] Moreover, they showed that NOX-101, a scavenger of NO, prevented endothelial dysfunction in STZ-induced diabetic rats.[39] However, it has also been shown that chronic treatment with an NO donor can ameliorate diminished responses to ACh in aorta and kidney vasculature caused by streptozotocin.[40] Whether the observed increase in basal NO activity shown in our model also causes microvascular damage in a later phase of the disease needs further elucidation.

In the control rats, the maximal responses to ACh were comparable to those found by other investigators using the same spinotrapezius muscle preparation.[15] In the diabetic rats of the present study, ACh still had the capacity to dilate the arterioles maximally after 6 weeks of diabetes. The maximal vasodilation caused by ACh could only partly be blocked by L-NNA, indicating that in this study the maximal response to ACh was mainly independent from nitric oxide production. Although ACh is often used as a means to measure stimulated NO-mediated vasodilatation it is well known that ACh not only releases NO, but also causes an increased production of the vasodilator endothelial derived hyperpolarizing factor (EDHF) and prostacyclin ($PGI_2$) as well as several vasoconstricting mediators. Streptozotocin diabetes can cause an increase in ACh-induced $PGI_2$ production.[40] As we focussed on possible alterations in NO production and sensitivity we cannot exclude that inside diameter differences between control and diabetic rats in this study are partly caused by changes in production of other vasorelaxing or constriciting agents.

After blocking endogenous NO production, we found a further difference between diabetic and control rats: a decrease in the effect of $10^{-4}$ mol/L nitroprusside in diabetic rats in the A2 arterioles. Nitroprusside, as a direct donor of NO to smooth muscle cells, is often used to establish whether the response of smooth muscle cells to NO is still intact. When the NP effect is impaired, a defect in the mechanism by which

NO stimulates the smooth muscle cells through activation of guanylate cyclase and production of cGMP is assumed.[41] The dose of nitroprusside we used ($10^{-4}$ mol/L), which was high enough to cause maximal dilatation in the arcade arterioles (A2) of control rats, was not able to cause maximal dilatation in the same order arterioles of diabetic rats. The cause of this phenomenon is presently unclear, but it is possible that the mechanism *via* which NO activates guanylate cyclase in smooth muscle cells is impaired after 6 weeks of STZ induced diabetes. An argument in favor of this hypothesis is the fact that A2 arterioles contain a relatively high amount of smooth muscle cells. An early increase in NO production may cause a decreased expression of guanylate cyclase or a desensitisation of guanylate cyclase.[42]

In this study we investigated changes in arteriolar reactivity after 6 weeks of STZ induced diabetes. We found an increased basal diameter in A3 and A4 arterioles and an unchanged diameter in A2 arterioles. This would be expected to result in a decreased pressure gradient from A2 arterioles to precapillary arterioles and thus in an elevated capillary pressure, which indeed has been observed in patients with type 1 diabetes mellitus.[43]

There are several possible causes for the changes in arteriolar reactivity in streptozotocin diabetes. Firstly, moderate hyperglycemia can cause changes in oxidative stress and increased production of reactive glycation products. Both for acute and chronic hyperglycemia it has been shown that hyperglycemia increases NO production and decreases the bioavailability of NO.[44,45] Secondly, changes in arteriolar reactivity might be primarily due to the decreased delivery of nutrients to the muscle cells, causing an autoregulatory dysfunction leading to vasodilatation.[2–4] Thirdly, streptozotocin-induced diabetes causes marked atrophy of hindlimb skeletal muscles, which is accompanied by a relative increase in flow capacity per tissue mass.[46] It cannot be excluded that the changes in arteriolar reactivity that were observed in this study might have been partly caused by an attenuation of muscle development that is caused by STZ diabetes, but that is also part of the clinical picture of type 1 diabetes. However, the exact mechanism *via* which STZ diabetes can cause the observed changes in arteriolar reactivity is beyond the scope of this study.

In conclusion, after 6 weeks of STZ-induced diabetes in rats, we found an increased basal diameter of small transverse arterioles of the spinotrapezius muscle but not of the larger arcade arterioles. There was a significantly increased contribution of endogenous NO to basal tone in A3 arterioles. The reactivity to exogenous NO was impaired in A2, but not in A3 and A4 arterioles. Thus, this study suggests a role of alterations in NO activity and sensitivity to NO in the pathogenesis of diabetic microvascular complications. Furthermore, our results show that these diabetes-induced changes are anatomically specific and, for arterioles, depend on their position within the vascular tree.

### Acknowledgements

This study was supported in part (JH Reitsma) by a grant from the Diabetes Fonds Nederland (Diabetes Research Fund #93.106). Dr. CDA Stehouwer was supported by a Clinical Research Fellowship from the Netherlands Organisation for Scientific Research (NWO). Prof. VWM van Hinsbergh is acknowledged for critically reading the manuscript. The authors thank Mr. W. Gerrissen for his biotechnical help.

### References

[1] Anonymous (1994). Microvascular and acute complications in IDDM patients: the EURODIAB IDDM Complications Study. *Diabetologia*, 37(3), 278–85.

[2] Tooke, J. E. (1986). Microvascular haemodynamics in diabetes mellitus. *Clinical Science*, 70(2), 119–25.

[3] Parving, H. H., Viberti, G. C., Keen, H., Christiansen, J. S. and Lassen, N. A. (1983). Hemodynamic factors in the genesis of diabetic microangiopathy. *Metabolism: Clinical and Experimental*, 32(9), 943–9.

[4] Zatz, R. and Brenner, B. M. (1986). Pathogenesis of diabetic microangiopathy. The hemodynamic view. *American Journal of Medicine*, **80**(3), 443–53.
[5] Vaughn, M. W., Kuo, L. and Liao, J. C. (1998). Estimation of nitric oxide production and reaction rates in tissue by use of a mathematical model. *American Journal of Physiology*, **274**(6 Pt. 2), H2163–H2176.
[6] Vaughn, M. W., Kuo, L. and Liao, J. C. (1998). Effective diffusion distance of nitric oxide in the microcirculation. *American Journal of Physiology*, **274**(5 Pt. 2), H1705–H1714.
[7] Pieper, G. M. (1998). Review of alterations in endothelial nitric oxide production in diabetes: protective role of arginine on endothelial dysfunction. *Hypertension*, **31**(5), 1047–60.
[8] Tooke, J. E. (1995). Microvascular function in human diabetes. A physiological perspective. *Diabetes*, **44**(7), 721–6.
[9] Muller, J. M., Davis, M. J. and Chilian, W. M. (1996). Integrated regulation of pressure and flow in the coronary microcirculation. *Cardiovascular Research*, **32**(4), 668–78.
[10] Jones, C. J., Kuo, L., Davis, M. J. and Chilian, W. M. (1995). Regulation of coronary blood flow: coordination of heterogeneous control mechanisms in vascular microdomains. *Cardiovascular Research*, **29**(5), 585–96.
[11] Davis, M. J. (1993). Myogenic response gradient in an arteriolar network. *American Journal of Physiology*, **264**(6 Pt. 2), H2168–H2179.
[12] Frame, M. D. and Sarelius, I. H. (1993). Regulation of capillary perfusion by small arterioles is spatially organized. *Circulation Research*, **73**(1), 155–63.
[13] Crijns, F. R., Struijker, B. H. and Wolffenbuttel, B. H. (1998). Arteriolar reactivity in conscious diabetic rats: influence of aminoguanidine treatment. *Diabetes*, **47**(6), 918–23.
[14] Gray, S. D. (1971). Effect of hypertonicity on vascular dimensions in skeletal muscle. *Microvascular Research*, **3**(1), 117–24.
[15] Nakamura, T. and Prewitt, R. L. (1991). Effect of NG-monomethyl-L-arginine on arcade arterioles of rat spinotrapezius muscles. *American Journal of Physiology*, **261**(1 Pt. 2), H46–H52.
[16] Tomlinson, K. C., Gardiner, S. M., Hebden, R. A. and Bennett, T. (1992). Functional consequences of streptozotocin-induced diabetes mellitus, with particular reference to the cardiovascular system. *Pharmacological Reviews*, **44**(1), 103–50.
[17] Heemskerk, A. E., Huisman, E., van, L. A., van dB, G. C., Hennekes, M. W., Thijs, L. G. and Tangelder, G. J. (1997). Influence of fluid resuscitation on renal function in bacteremic and endotoxemic rats. *Journal of Critical Care*, **12**(3), 120–31.
[18] Heemskerk, A. E., Huisman, E., van, L. A., van dB, G. C., Hennekes, M., Thijs, L. G. and Tangelder, G. J. (1997). Renal function and oxygen consumption during bacteraemia and endotoxaemia in rats. *Nephrology, Dialysis, Transplantation*, **12**(8), 1586–94.
[19] Marshall, J. M. (1982). The influence of the sympathetic nervous system on individual vessels of the microcirculation of skeletal muscle of the rat. *Journal of Physiology*, **332**, 169–86.
[20] Engelson, E. T., Skalak, T. C. and Schmid-Schonbein, G. W. (1985). The microvasculature in skeletal muscle. I. Arteriolar network in rat spinotrapezius muscle. *Microvascular Research*, **30**(1), 29–44.
[21] Skalak, T. C. and Schmid-Schonbein, G. W. (1986). The microvasculature in skeletal muscle. IV. A model of the capillary network. *Microvascular Research*, **32**(3), 333–47.
[22] Van den Bos, G. C., Westerhof, N., Hoogerwerf, N. and Sicking, A. H. (1991). Arteriolar and venular reactivity to superfusate $pO_2$ in tissues with different metabolic capacity. A study in skeletal muscle and mesentery of the rat. *International Journal of Microcirculation: Clinical and Experimental*, **10**(4), 303–16.
[23] Rongen, G. A., Floras, J. S., Lenders, J. W., Thien, T. and Smits, P. (1997). Cardiovascular pharmacology of purines. *Clinical Science*, **92**(1), 13–24.
[24] Rongen, G. A., Ginneken, E., Thien, T., Lutterman, J. A. and Smits, P. (1996). Preserved vasodilator response to adenosine in insulin-dependent diabetes mellitus. *European Journal of Clinical Investigation*, **26**(3), 192–8.
[25] Rongen, G. A., Lenders, J. W., Lambrou, J., Willemsen, J. J., van Belle, H., Thien, T. and Smits, P. (1996). Presynaptic inhibition of norepinephrine release from sympathetic nerve endings by endogenous adenosine. *Hypertension*, **27**(4), 933–8.
[26] Lash, J. M. and Bohlen, H. G. (1991). Structural and functional origins of suppressed acetylcholine vasodilation in diabetic rat intestinal arterioles. *Circulation Research*, **69**(5), 1259–68.
[27] Kiff, R. J., Gardiner, S. M., Compton, A. M. and Bennett, T. (1991). Selective impairment of hindquarters vasodilator responses to bradykinin in conscious Wistar rats with streptozotocin-induced diabetes mellitus. *British Journal of Pharmacology*, **103**(2), 1357–62.
[28] Williamson, J. R., Chang, K., Frangos, M., Hasan, K. S., Ido, Y., Kawamura, T., Nyengaard, J. R., van, dE, Kilo, C. and Tilton, R. G. (1993). Hyperglycemic pseudohypoxia and diabetic complications. *Diabetes*, **42**(6), 801–13.
[29] Komers, R., Allen, T. J. and Cooper, M. E. (1994). Role of endothelium-derived nitric oxide in the pathogenesis of the renal hemodynamic changes of experimental diabetes. *Diabetes*, **43**(10), 1190–7.
[30] Oyama, Y., Kawasaki, H., Hattori, Y. and Kanno, M. (1986). Attenuation of endothelium-dependent relaxation in aorta from diabetic rats. *European Journal of Pharmacology*, **132**(1), 75–8.
[31] Kamata, K., Miyata, N. and Kasuya, Y. (1989). Impairment of endothelium-dependent relaxation and changes in levels of cyclic GMP in aorta from streptozotocin-induced diabetic rats. *British Journal of Pharmacology*, **97**(2), 614–8.
[32] Pieper, G. M. and Gross, G. J. (1988). Oxygen free radicals abolish endothelium-dependent relaxation in diabetic rat aorta. *Am. J. Physiol.*, **255**(4 Pt. 2), H825–H833.
[33] Oyama, Y., Kawasaki, H., Hattori, Y. and Kanno, M. (1986). Attenuation of endothelium-dependent relaxation in aorta from diabetic rats. *Eur. J. Pharmacol.*, **132**(1), 75–8.
[34] Moncada, S., Palmer, R. M. and Higgs, E. A. (1991). Nitric oxide: physiology, pathophysiology, and pharmacology. *Pharmacological Reviews*, **43**(2), 109–42.
[35] Harrison, D. G. (1997). Cellular and molecular mechanisms of endothelial cell dysfunction. *Journal of Clinical Investigation*, **100**(9), 2153–7.

[36] Wever, R. M., Luscher, T. F., Cosentino, F. and Rabelink, T. J. (1998). Atherosclerosis and the two faces of endothelial nitric oxide synthase. *Circulation*, **97**(1), 108–12.

[37] Munzel, T., Heitzer, T. and Harrison, D. G. (1997). The physiology and pathophysiology of the nitric oxide/superoxide system. *Herz*, **22**(3), 158–72.

[38] Pieper, G. M. (1999). Enhanced, unaltered and impaired nitric oxide-mediated endothelium-dependent relaxation in experimental diabetes mellitus: importance of disease duration. *Diabetologia*, **42**(2), 204–13.

[39] Pieper, G. M., Dembny, K. and Siebeneich, W. (1998). Long-term treatment *in vivo* with NOX-101, a scavenger of nitric oxide, prevents diabetes-induced endothelial dysfunction. *Diabetologia*, **41**(10), 1220–6.

[40] Kamata, K. and Hosokawa, M. (1997). Endothelial dysfunction in the perfused kidney from the streptozotocin-induced diabetic rat. *Res. Commun. Mol. Pathol. Pharmacol.*, **96**(1), 57–70.

[41] Murad, F. (1986). Cyclic guanosine monophosphate as a mediator of vasodilation. *Journal of Clinical Investigation*, **78**(1), 1–5.

[42] Brandes, R. P., Kim, D., Schmitz-Winnenthal, F. H., Amidi, M., Godecke, A., Mulsch, A. and Busse, R., Increased nitrovasodilator sensitivity in endothelial nitric oxide synthase knockout mice: role of soluble guanylyl cyclase. *Hypertension*, January, 2000, **35**.(1.Pt.2.), 231.–6. **35**(1 Pt. 2), 231–6.

[43] Sandeman, D. D., Shore, A. C. and Tooke, J. E. (1992). Relation of skin capillary pressure in patients with insulin-dependent diabetes mellitus to complications and metabolic control. *New England Journal of Medicine*, **327**(11), 760–4.

[44] Graier, W. F., Posch, K., Wascher, T. C. and Kostner, G. M. (1997). Role of superoxide anions in changes of endothelial vasoactive response during acute hyperglycemia. *Horm. Metab. Res.*, **29**(12), 622–6.

[45] Claxton, C. R., Brands, M. W., Fitzgerald, S. M. and Cameron, J. A., Inhibition of nitric oxide synthesis potentiates hypertension during chronic glucose infusion in rats. *Hypertension*, January, 2000, **35**.(1.Pt.2.), 451.–6. **35**(1 Pt. 2), 451–6.

[46] Sexton, W. L. (1994). Skeletal muscle vascular transport capacity in diabetic rats. *Diabetes*, **43**(2), 225–31.

